# ROLE OF BONE GRAFTS AND BONE GRAFT SUBSTITUTES IN ISOLATED SUBTALAR JOINT ARTHRODESIS

**DOI:** 10.1590/1413-785220172505177665

**Published:** 2017

**Authors:** ASHISH SHAH, SAMEER NARANJE, IBUKUNOLUWA ARAOYE, OSAMA ELATTAR, ALEXANDRE LEME GODOY-SANTOS, CESAR DE CESAR

**Affiliations:** 1. Department of Surgery, Division of Orthopedic Surgery, University of Alabama, Birmingham, AL, USA.; 2. Department of Orthopedic Surgery, Forrest City Medical Center, Forrest City, AR, USA.; 3. Orthopedic Sports Medicine Service, Boston University, Boston, MA, USA.; 4. Instituto de Ortopedia e Traumatologia, Hospital das Clínicas, Faculdade de Medicina, Universidade de São Paulo, São Paulo, SP, Brazil.

**Keywords:** Arthrodesis, Bone transplantation, Calcaneus, Subtalar joint, Transplantation, homologous

## Abstract

**Objectives:**

The purpose of this study was to compare union rates for isolated subtalar arthrodesis with and without the use of bone grafts or bone graft substitutes.

**Methods:**

We retrospectively reviewed 135 subtalar fusions with a mean follow-up of 18 ± 14 months. The standard approach was used for all surgeries. Graft materials included b-tricalcium phosphate, demineralized bone matrix, iliac crest autograft and allograft, and allograft cancellous chips. Successful subtalar fusion was determined clinically and radiographically.

**Results:**

There was an 88% (37/42) union rate without graft use and an 83% (78/93) union rate with bone graft use. Odds ratio of union for graft versus no graft was 0.703 (95% CI, 0.237-2.08). The average time to union in the graft group was 3 ± 0.73 months and 3 ± 0.86 in the non-graft group, with no statistically significant difference detected (p = 0.56).

**Conclusion:**

Graft use did not improve union rates for subtalar arthrodesis. **Level of Evidence IV, Case Series.**

## RESUMO

**Objetivos:**

O propósito deste estudo foi comparar as taxas de união de artrodese subtalar isolada com e sem uso de enxertos ósseos ou seus substitutos.

**Métodos:**

Revisamos retrospectivamente 135 fusões subtalares com seguimento médio de 18 ± 14 meses. A via de acesso padrão foi utilizada em todas as cirurgias. Os enxertos utilizados incluíram fosfato b-tricálcico, matriz óssea desmineralizada, autoenxerto e aloenxertos da crista ilíaca e aloenxerto de lascas de osso trabecular. A fusão subtalar bem-sucedida foi determinada clínica e radiograficamente.

**Resultados:**

Verificou-se uma taxa de união de 88% (37/42) sem uso de enxerto e de 83% (78/93) com enxerto ósseo. A análise da razão de chances (odds ratio) de união óssea para enxerto e não enxerto foi 0,703 (IC 95%, 0,237-2,08). O tempo médio de união no grupo com enxerto foi de 3 ± 0,73 meses e 3 ± 0,86 no grupo sem enxerto, sem detecção de diferença estatisticamente significante (p = 0,56).

**Conclusão:**

O uso de enxerto não melhorou as taxas de união na artrodese subtalar. **Nível de Evidência IV, Série de Casos.**

## INTRODUCTION

Subtalar joint (STJ) arthrodesis is a well-established operative procedure in the treatment of subtalar arthritis and hindfoot deformities. Indications include primary degenerative arthritis, inflammatory arthritis, post-infectious arthritis, congenital hindfoot deformities, talocalcaneal coalitions, and posterior tibial tendon dysfunction. The main goals of STJ arthrodesis are pain relief, hindfoot realignment, and functional improvement.^1^ ^-^ ^3^ Traditionally, triple arthrodesis has been the operative gold standard for resistant talocalcaneal pathologies but, more recently, isolated STJ arthrodesis has seen increased advocacy. Suggested advantages of the isolated approach include simpler and shorter operations, lower risk of transverse tarsal joint nonunion or mal-union, and preservation of some hindfoot motion.^3^

Nonunion remains an important complication, with incidence and role of risk factors varying in the literature. Recent reports have highlighted a decrease in overall union rates from between 96% and 100% to 84%^3^ ^,^ ^4^ further strengthening the need for an understanding of risk factors that may be implicated in nonunion rates. Some possible factors have been identified including smoking, revision surgery, the presence and extent of devascularized bone, and previous ankle joint fusion.^4^ Operative technique may represent another factor especially with regard to the degree of compression and the rigidity achieved at the fusion site.^5^

The purpose of this retrospective study was to compare the fusion rates (both clinically and radiographically) and the time to union of STJ arthrodesis with and without the use of concomitant bone grafting. We hypothesized that the use of bone grafts or bone graft substitutes would not improve union rates and time to union. We also evaluated the association of smoking and the occurrence of STJ nonunion.

## MATERIALS AND METHODS 

We reviewed the charts and radiographs of 133 patients who underwent 135 primary STJ arthrodesis between January 2010 and December 2013, after the approval of Research Ethics Committee of our Institution (IRB number: X160503004). There were 66 males and 67 females. Mean age was 48 (range, 18 to 74) years. Forty-one cases (feet) were smokers (26 in the graft group and 15 in the non-graft group) and 19 cases (feet) were diabetics (10 in the graft group and 9 in the non-graft group). (Table 1) Patients with concomitant or prior foot and ankle fusions, revision subtalar fusion, concomitant total ankle replacement, or distraction arthrodesis were excluded. Primary diagnoses included flat foot secondary to posterior tibial tendon dysfunction (44 feet), post-traumatic osteoarthrosis (41 feet), primary osteoarthrosis (no other specific diagnosis made) (29 feet), tarsal coalition (11 feet), inflammatory (e.g. rheumatoid) joint disease (6 feet), and neurological disorders with STJ instability (4 feet). 

**Table 1:** Patient group demographics with group comparison *p* values (n = 135 feet).

	Total (n=135 feet)	Graft (n=93 feet)	Nongraft (=42 feet)	*p* value
Gender				0.35
Male	66	48	18	
Female	67	43	24	
**Mean age** (years)	48±15	47±16	51±14	**0.21**
**Tobbaco use**				**0.42**
Smoker	41	26	15	
Nonsmoker	94	67	27	
**Diabetes**				
Diabetic	19	10	9	**0.11**
Nondiabetic	116	83	33	
**Mean follow-up** (months)	18±14	16±13	23±14	**0.01**
**Screws**				
Single	16	3	13	**<0.001**
Double	119	90	29	**0.02**
Parallel	11	5	6	
Divergent	108	85	23	

Values are given as absolutely number or as mean ± SD with p values for the Fisher’s exact test (significance declared when p < .05).

All patients were evaluated clinically and radiographically (AP, lateral, and subtalar views) until union was achieved or the diagnosis of nonunion was established by CT. Clinically, fusion was defined by subtalar joint stability in the absence of symptoms. Radiographically, fusion was defined as obliteration of the joint space with the presence of crossing trabeculae. CT criteria for fusion was consolidation of at least 50% of the posterior facet of the subtalar joint. All suspected cases of delayed union or nonunion were evaluated by CT.

Patients were divided into one of two operative groups - graft group or non-graft group - for comparison of the primary outcome of interest (union rate) using Fisher’s exact test. Secondarily, Fisher’s exact test was used in comparing the union rates in smokers and nonsmokers. Logistic regression was also used to compare odds of union for graft versus non-graft and smoker versus nonsmoker. All statistical analyses were performed on SPSS 23.0 software (IBM Corporation, New York, NY, USA) with significance level set at *p* < 0.05.

### Operative technique 

**Figure 1:**
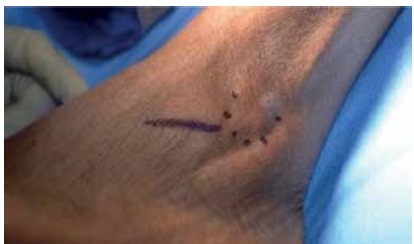
Clinical photograph showing the standard lateral surgical approach to the subtalar joint (sinus tarsi incision).

**Figure 2:**
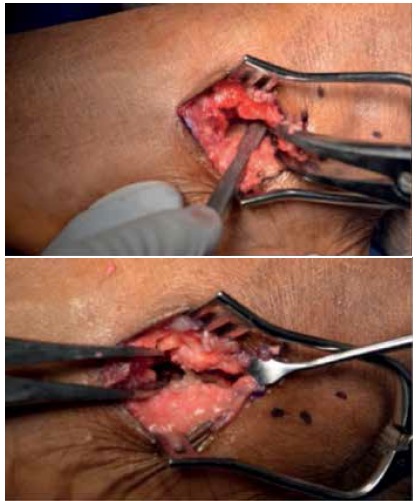
Intraoperative photograph showing preparation of the subtalar joint using the fish-scaling technique.

Patients were draped and prepped (including a thigh tourniquet) in a sterile fashion. Skin incision and joint exposure were performed as described, (Figure 1) until the flexor hallucis longus (FHL) tendon was visible medially. This was followed by either drilling (for the graft group) or fish-scaling (for the nongraft group) (Figure 2) of the subchondral bone to promote healing/fusion post-fixation. Joint apposition was assessed and then followed by either bone grafting or screw fixation. Bone graft was used in 93 feet while bone graft was not used in 42 feet. Decision to graft or not to graft was based solely on surgeon preference. Graft types used included b-tricalcium phosphate (b-TCP) mixed with bone marrow aspirate (proximal tibia) (82 feet); demineralized bone matrix (DBM) mixed with bone marrow aspirate (proximal tibia) (8 feet); iliac crest autograft (2 feet); and allograft cancellous chips (1 foot).

**Figure 3:**
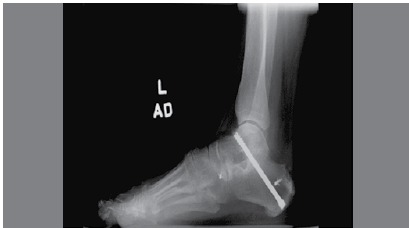
Postoperative plain lateral ankle radiograph showing solid union at the subtalar joint arthrodesis site using one 7.3mm lag screw.

Once bony apposition was achieved with proper hindfoot alignment, K-wires were inserted from the calcaneal tuberosity into the talar dome across the posterior facet. Positioning was confirmed fluoroscopically. With satisfactory positioning confirmed, definitive fixation was achieved using either a single 7.3mm screw (16 feet) or two 6.5mm screws (119 feet) (the gold standard for STJ arthrodesis). (Figure 3) The screws were partially threaded cancellous screws. Single-screw fixation was performed in the talocalcaneal direction. Double-screw fixation was performed in either a parallel (11 feet) or divergent fashion (108 feet) with talocalcaneal direction in 96 feet, calcaneotalar direction in 20 feet, and mixed direction (1 talocalcaneal, 1 calcaneotalar) in 3 feet. 

### Postoperative care 

**Figure 4:**
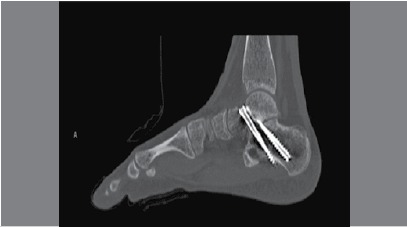
Postoperative sagittal CT scan cut showing nonunion at the subtalar joint arthrodesis site.

The splint was removed at 2 weeks for a wound check and stitch removal. This was followed by 6 weeks of non-weight-bearing cast use, and then removable boot cast use until clinical and radiographic confirmation of healing/fusion. Assessment of healing/fusion was performed clinically and radiographically every 6 weeks. Patients were followed for a mean of 18 ± 14 months. Poor union was determined as the presence of persistent pain and tenderness as well as poor radiographic evidence of progressive healing (i.e. lack of trabeculae across the fusion site). Patients with residual symptoms by week 16 to 20 postoperatively were evaluated by CT (Figure 4) and were then either confirmed as nonunions or explained by other pathology.

## RESULTS

There was an overall union rate of 85% (115/135) and CT-confirmed nonunion rate of 15% (20/135). (Table 2) The average time to union was 3 ± 1 months. There was an 88% (37/42) union rate without graft compared to an 83% (78/93) union rate with bone graft use (TCP = 69/80; DMB = 7/8; iliac crest autograft = 1/2; allograft cancellous chips = 1/1). (Table 3) Union rate was not significantly different between the graft and nongraft groups (*p* = 0.61). Odds ratio of union for graft versus non-graft was 0.703 (95% CI, 0.237 - 2.08). The average time to union in the graft group was 3 ± 0.73 months and 3 ± 0.86 in the nongraft group with no statistically significant difference detected (*p* = 0.56). 

**Table 2:** Union rate as a function of graft status, smoking status, diabetes status, number of screws, and screw orientation (for double screws) (n = 135 feet).

	Union rate	*p* value
Grafs status		
Graft	83 (78/93)	0.35
Nongraft	88 (37/42)	
**Tobbaco use**		
Smoker	78 (32/41)	0.19
Nonsmoker	88 (83/94)	
**Diabetes**		
Diabetic	100 (1/1)	0.08
Nondiabetic	83 (96/116)	
**Screws**		
Single	94 (15/16)	0.46 0.69
Double	84 (100/119)	
Parallel	82 (9/11)	
Divergent	84 (91/108)	

Values are given as percentage (absolutely number in parentheses) with p values for the Fisher’s exact test (significance declared when p < .05).

**Table 3:** Union rate by graft type, a = b-tricalcium phosphate, b = demineralized bone matric (n = 93 feet).

Graft type	Union rate
b-TCP^a^ + bone marrow aspirate (proximal tibia)	84 (69/82)
DBM^b^ + bone marrow aspirate (proximal tibia)	88 (7/8)
Iliac crest autograft	50 (1/2)
Allograft cancellous chips	100 (1/1)
Total	84 (78/93)

Values are given as percentage (absolutely number in parentheses).

There were 41 (feet) smokers in the study population (26 in the graft group, 15 in the non-graft group). The smoking population had a 78% (32/41) union rate compared to an 88% (83/94) union rate in nonsmokers. However, these union rates were not significantly different between smokers and nonsmokers (*p* = 0.19). (Table 2) The odds ratio of union for smokers versus nonsmokers was 0.471 (95% CI, 0.178 - 1.244). Graft smokers had a union rate of 73% (19/26) while non-graft smokers had a union rate of 87% (13/15) without being significantly different from each other (*p* = 0.45). Excluding smokers from data analysis resulted in a union rate of 88% (83/94) (85% (115/135) when included).

All of the 19 diabetic patients included in this study achieved union (10 in graft group, 9 in nongraft group). (Table 2) There were 6 patients with rheumatoid arthritis (RA) (4 in graft group, 2 in non-graft group). Only one RA patient did not achieve union and this patient was from the graft group. There was one leukemia patient on chemotherapy and this patient achieved union.

Complications included: 7 deep infections (3 required irrigation and debridement along with intravenous antibiotics); 1 wound dehiscence (resolved with wound care); 5 sural neuritis cases (2 required nerve block or neurectomy. Others self-resolved); 3 complex regional pain syndrome cases; 1 talar neck stress fracture (between 2 screws) (conservatively managed); 5 persistent pain cases requiring hardware removal; 2 subfibular impingements (1 required arthroscopic debridement. The other was managed non-operatively). The possible risk factors for nonunion cases are described in Table 4.

**Table 4:** Possible risk factors for nonunion (n = 20 feet).

Number of nonunions	Possible risk factor
6	Posttraumtic (2 were smokers in addition)
6	Smoker (only risk factor)
2	Talar AVN (1 posttraumatic, 1 RA and smoker)
6	None

Abbreviations: AVN - avascular necrosis, RA - rheumatoid arthritis.

## DISCUSSION

Despite the recent trends towards minimally invasive operative techniques and the increasing use of subtalar arthroscopic fusion,^6^ the open approach is still preferred for STJ arthrodesis. Many options for fixation of the arthrodesis have been described including staples,^7^ dowels,^8^ and lag screws.^9^ However, screw fixation remains the gold standard. Regardless of the number, size or directionality (calcaneotalar or talocalcaneal) of the lag screw in fixation, union rates ranging from 86% to 100% have been reported.^4^ ^,^ ^7^ ^-^ ^9^ Of note, studies with a large *n* (ranging from 95 to 184 feet) usually report union rates (85%-90%) closer to what we find in our study (85%).^4^ ^,^ ^9^ On the other hand, studies finding union rates close to 100% are usually small n studies (ranging from 19 to 48 feet) (some due to significant losses in patient follow-up).^7^ Although using 101 feet, Haskell et al.^9^ reported a 98% union rate.

Compared to other bone graft types, autogenous bone grafts carry a lower risk of infection transmission and are more likely to incorporate at their new site since they have minimal to no immunogenicity.^10^ Nonetheless, autogenous bone grafting carries significant disadvantages including donor site pain and morbidity (even with the new trapdoor harvesting technique).^11^ It is also associated with prolonged operating room time (especially in nonacademic settings where the option of two surgical teams is not always available), greater blood loss, and increased postoperative pain. In addition, greater cost is incurred in cases where additional surgery is required to obtain the bone grafts.^12^

In the past decade, there has been a revolutionary change in the array of bone grafting products available^10^ ^,^ ^11^ with allografts being the first alternative to autografts. Subsequently, demineralized bone matrix (DBM) was developed and became a viable substitute for allografts as an alternative to autogenous bone grafts (24-31). It has good osteoinductive properties due to release of growth factors during the demineralization process - however the sterilization process slightly decreases these osteoinductive properties.^12^ When preparing DBM for implantation, it is usually mixed with bone marrow, increasing possible osteogenic factors and pluripotent cells. It can also be used as an autogenous bone graft expander.^13^

The emergence of new synthetic bone graft products has been of great interest to the orthopedic community during the last decade.^12^ Synthetic bone graft materials offer an effective alternative to autografts, allografts, and demineralized bone matrix. An example of synthetic bone graft material is b-TCP, which is sterilely prepared, osteoconductive, and highly effective in filling bone void defects of the extremities.^13^ When prepared with bone marrow, b-TCP provides an excellent osteoconductive structure, with osteogenic capabilities from the marrow.^12^

Scranton recommends bone grafting to avoid nonunion^14^ whereas Kitaoka and Patzer^15^ and Tasto^16^ achieved 100% union without bone grafting, concluding that bone grafting is not necessary for obtaining joint fusion. Dahm and Kitaoka^17^ also concluded that bone grafting is not essential for achieving union in STJ arthrodesis (although this was in patients following intraarticular calcaneal fracture). Joveniaux et al.^18^ evaluated patients undergoing subtalar arthrodesis by grafting and found no statistically significant difference in time to union between patients with and without grafting in terms of union time. Moreover, the four revision arthrodeses in their series did not receive bone grafting during the first procedure.^18^ To our knowledge, no large studies have been published to specifically compare fusion outcomes (union rates and time to union) in graft-supported STJ arthrodesis to fusion outcomes in non-grafted STJ arthrodesis. 

With respect to our union rate findings for each graft type, it is difficult to make any conclusions due to scant sample sizes for each graft type except b-TCP. b-TCP, when prepared with bone marrow, provides an excellent osteoconductive structure, with osteogenic capabilities from the marrow.^12^ In our study, b-TCP synthetic bone graft mixed with bone marrow aspirate was used in 82 feet with a union rate of 84% (69/82). Although, the small sample sizes for each other graft types are sub-optimal, union rates were not alarmingly different from current literature findings. Michelson and Curl^12^ conducted a prospective study comparing autogenous iliac crest bone graft to DBM in 55 patients undergoing hindfoot arthrodesis, finding no significant difference in healing between the two groups of bone graft patients. Similarly, there was no significant difference in the time to healing between the iliac crest bone graft fusions and the DBM fusions. In their patient series, DBM was used in 36 hindfoot fusions with union achieved in 35 feet (97.2%).^12^ Our study found a DBM union rate of 88% (7/8). As reports of DBM use in foot and ankle surgery are limited, it represents an area where more studies will be beneficial. 

Easley et al.^4^ reported a 92% union rate in nonsmokers versus a 73% union rate in smokers (*p* < .01). Similarly, Ishikawa et al.^19^ found that smokers were 2.7 times more likely to have a nonunion when compared to non-smokers. Despite such evidence in the literature for the association of smoking with nonunion, our study fails to replicate this finding. Possible explanations may include the weakness of the effect of smoking on union rates as well as sampling bias associated with retrospective studies. Particularly, the decision to graft or not may have been influenced by intraoperative findings or implicitly by smoking status.

Our study had several limitations. To begin with, it is a retrospective study based on reviewing patients’ clinical charts and radiographs, limiting information such as patient outcome scores. In the same vein, other issues associated with the lack of variable control in retrospective studies are also noted in this study. For example, we found that patients in the non-graft group were more likely to have single screw fixation when compared to patients in the graft group. Of note however, this finding reflected the inclination of a single surgeon for both single screw and non-grafted operative technique (almost all single screw cases were performed by this surgeon). Another weakness of this study is the fact that successful fusion was entirely based on clinical judgment supplemented by radiographic evidence of healing. CT scan was not obtained for every patient to confirm union. While this would be ideal, this would expose a large number of patients to unnecessary expense and radiation. Because these patients had no pain on weight bearing and their plain radiographs confirmed union, a CT scan was not thought necessary. 

## CONCLUSION

The use of bone graft or bone graft substitutes in STJ arthrodesis did not result in higher fusion rates nor did they shorten the time to union when compared to STJ arthrodesis without graft use. In addition, smoking status did not negatively impact union outcome. 

## References

[1] Davies MB, Rosenfeld PF, Stavrou P, Saxby TS (2007). A comprehensive review of subtalar arthrodesis. Foot Ankle Int.

[2] Diezi C, Favre P, Vienne P (2008). Primary isolated subtalar arthrodesis outcome after 2 to 5 years follow-up. Foot Ankle Int.

[3] Mann RA, Beaman DN, Horton GA (1998). Isolated subtalar arthrodesis. Foot Ankle Int.

[4] Easley ME, Trnka HJ, Schon LC, Myerson MS (2000). Isolated subtalar arthrodesis. J Bone Joint Surg Am.

[5] Hintermann B, Valderrabano V, Nigg B (2002). Influence of screw type on obtained contact area and contact force in a cadaveric subtalar arthrodesis model. Foot Ankle Int.

[6] Glanzmann MC, Sanhueza-Hernandez R (2007). Arthroscopic subtalar arthrodesis for symptomatic osteoarthritis of the hindfoot a prospective study of 41 cases. Foot Ankle Int.

[7] Chandler JT, Bonar SK, Anderson RB, Davis WH (1999). Results of in situ subtalar arthrodesis for late sequelae of calcaneus fractures. Foot Ankle Int.

[8] Dennyson WG, Fulford GE (1976). Subtalar arthrodesis by cancellous grafts and metallic internal fixation. J Bone Joint Surg Br.

[9] Haskell A, Pfeiff C, Mann R (2004). Subtalar joint arthrodesis using a single lag screw. Foot Ankle Int.

[10] Bostrom M, Seigerman D (2005). The clinical use of allografts, demineralized bone matrices, synthetic bone graft substitutes and osteoinductive growth factors a survey study. HSS J.

[11] Keating JF, McQueen MM (2001). Substitutes for autologous bone graft in orthopaedic trauma. J Bone Joint Surg Br.

[12] Michelson J, Curl L (1996). Use of demineralized bone matrix in hindfoot arthrodesis. Clin Orthop Relat Res.

[13] Sauer ST, Marymont JV, Mizel MS (2004). What&apos;s new in foot and ankle surgery. J Bone Joint Surg Am.

[14] 1Scranton PE (1991). Results of arthrodesis of the tarsus talocalcaneal, midtarsal, and subtalar joints. Foot Ankle.

[15] Kitaoka HB, Patzer GL (1997). Subtalar arthrodesis for posterior tibial tendon dysfunction and pes planus. Clin Orthop Relat Res.

[16] Tasto JP, Arthrodesis S (2006). Arthroscopy of the subtalar joint and arthroscopic subtalar arthrodesis. Instr Course Lect.

[17] Dahm DL, Kitaoka HB (1998). Subtalar arthrodesis with internal compression for post-traumatic arthritis. J Bone Joint Surg Br.

[18] Joveniaux P, Harisboure A, Ohl X, Dehoux E (2010). Long-term results of in situ subtalar arthrodesis. Int Orthop.

[19] Ishikawa SN, Murphy GA, Richardson EG (2000). The effect of cigarette smoking on hindfoot fusions. Foot Ankle Int.

